# Editorial: Soluble immune checkpoints: Novel physiological immunomodulators

**DOI:** 10.3389/fimmu.2023.1178541

**Published:** 2023-03-21

**Authors:** Antonio Riva

**Affiliations:** ^1^ The Roger Williams Institute of Hepatology, Foundation for Liver Research, London, United Kingdom; ^2^ Faculty of Life Sciences and Medicine, King’s College London, London, United Kingdom

**Keywords:** immune checkpoint, soluble immune checkpoint, immunotherapy, human disease, immunomodulation, novel therapeutic agents

Immune checkpoints are a vast and diverse array of stimulatory and inhibitory pathways that fine-tune immune responses. Molecules such as PD-1, CTLA4 and their ligands have gained attention as anti-cancer immunotherapy targets, and other checkpoint pathways have also been implicated in the immunopathogenesis of human disease (1–3). Immune checkpoints were traditionally assumed to be exclusively cell membrane-bound systems of receptors and ligands capable of switching on or off immune cell functions, but it is now clear that they also exist as soluble forms (soluble checkpoint receptors, or solCRs). These soluble molecules are not simply by-products of proteolytic degradation but are biologically active regulators that participate in the paracrine and systemic modulation of immune responses, similar to stimulatory or inhibitory cytokines.

While the association between solCRs and human disease is well established, the purpose of this Research Topic was to collect evidence on key aspects of soluble checkpoint biology that are still unclear:

1) molecular mechanism(s) responsible for their generation (alternative splicing, membrane ectodomain shedding, proteolytic cleavage);

2) existence of different isoforms and whether they can be detected individually;

3) balance between different isoforms in health and disease, and whether solCR profiles are disease-specific;

4) biological activity and whether it matches that of the respective membrane-bound form(s);

5) molecular mechanism(s) of action (binding partners, signal transduction, formation of soluble complexes between soluble receptor and soluble ligands, molecular decoys, etc.);

6) immunomodulatory functions of recombinant solCRs.

With particular emphasis on human studies, because the translational aspects of this research should be focused on human physiology and human disease, these molecular details may have crucial pathophysiological implications and may inform the therapeutic use of solCRs as novel immunomodulatory targets or pharmacological agents.

Landeira-Viñuela et al. describe significant differences in solCR profiles in chronic lymphocytic leukaemia patients (CLL). The authors perform an in-depth immune characterisation, measuring levels of over 100 soluble immune factors, and use advanced statistical methods to create decision trees to differentiate between disease stages, degrees of disease severity, and treatment response classes. Comprehensive molecular network assessments are then presented on the interplay of solCRs, cytokines, chemokines, and other factors in relation to differences in disease profiles, response to treatment, and mutational status.

Wang et al. investigate the role of solCRs in the progression and severity of non-small cell lung cancer (NSCLC). The authors establish the role of solCD27 and solPD-L2 in facilitating tumour invasiveness by quantifying solCRs in the blood of NSCLC patients and correlating these measurements with tissue-based expression data from publicly available datasets (here, “The Cancer Genome Atlas”, TCGA). The authors seek a direct link between these soluble molecules and the tumour microenvironment as a surrogate for their biological importance in cancer. Valid hypotheses on the mechanism of action of solCD27 and solPD-L2 as possible immune antagonists are then offered based on what is known about their membrane-bound forms.

Niu et al. provide an in-depth examination of solPD-1 and solPD-L1 and their roles in cancer. The review is dense with information that touches on many of the points that this Research Topic sought to clarify in the first place. The authors describe the molecular mechanisms underlying the synthesis and release of solPD-1 and solPD-L1, with alternative mRNA splicing and translation of isoforms lacking transmembrane domains being the most important. The authors discuss the complexities of multiple soluble isoforms and which isoforms may have biological activity. Studies linking the detection of these soluble molecules with specific immune alterations reveal that solPD-1 may function as an immune stimulator, in contrast to membrane-bound PD-1, whereas solPD-L1 retains suppressive activity. The possibility that solPD-1 and solPD-L1 may also act as decoy sponges for therapeutic blocking antibodies is advanced, with clear consequences in relation to resistance to immunotherapy and disease progression. Finally, the presence of biologically active exosomal PD-L1 and its role in immune suppression is highlighted, raising the possibility that other solCRs may circulate through exosomes and extracellular vesicles, blurring the line between membrane-bound and fully cell-free soluble isoforms.

Noubissi Nzeteu et al. investigate the biology of the T-cell inhibitory checkpoint VISTA during monocyte-to-macrophage differentiation and maturation. This paper elegantly demonstrates that immune cell differentiation, maturation and activation may be accompanied by considerable changes in solCR production and release. In this investigation, solVISTA declines progressively as monocytes develop into macrophages and then into activated M1 or M2 subsets. Changes caused by TLR4 signalling may be important for modulating antibacterial immunity, especially as solVISTA appears to have T-cell suppressive activity. Overall, this study suggests that solVISTA may be a physiological mechanism necessary for the maintenance and modulation of peripheral tolerance, and it sheds new light on the physiology of this immune checkpoint pathway.

Li et al. discuss the impact of solCRs in COVID-19 patients and HIV-infected alcohol users, providing new information on how these molecules may alter disease severity. The authors report a solCR ‘storm’ in COVID-19 patients and address how heavy alcohol use may exacerbate solCR abnormalities already existing in HIV patients, some of which may persist even after immune reconstitution with anti-retroviral therapy. After our first report of solCR network alterations and links with disease severity in patients with alcohol-related liver disease (4, 5), various papers have confirmed and expanded our findings (6–8), including the initial hypothesis that these molecules could become useful and effective immunotherapeutic agents for immune modulation. According to Li et al., HIV infection in heavy alcohol users can exacerbate these dysfunctions. The authors emphasise the physiological presence of solCRs in healthy subjects and explain how their balance is skewed in cancer, COVID-19, HIV, and other diseases. The authors also confirm that results obtained from different quantification kits are only partially overlapping, which has implications for data replicability and comparability between studies.

Ultimately, these publications help to answer some of the preliminary key questions we posed. The molecular mechanisms leading to synthesis of certain solCRs are now relatively well understood, including data on the generation of different isoforms. Different soluble isoforms may have different protein domains, which may influence biological activities such as the ability to bind respective ligands or receptors. This has significant implications for current immunotherapy, as the idea that solCRs may operate as decoys for therapeutic blocking antibodies may have a considerable impact on resistance to immunotherapy and disease progression.

SolCR networks are extensively intercorrelated, but despite this internal redundancy nuanced disease-specific signatures seem to exist, some of which persist even after pharmacological treatment or disease resolution as a sort of solCR “imprinting”. This could be important to gain more insight into disease mechanisms, the role of immune modulation in disease processes, and the perpetuation of possible risk factors derived from earlier diseases or treatments. Disease-specific profiles could also point to distinct approaches to pharmacological targeting and intervention.

The articles published in this Research Topic support the hypothesis that soluble isoforms of immune checkpoints may not always perform the same function as their membrane-bound counterparts (1), as demonstrated by the case of solPD-1 addressed here by Niu et al., or solTIM3 in our studies (4, 5). Although both membrane-bound PD-1 and TIM3 are immune inhibitors, experimental evidence suggests that their soluble isoforms are more likely to act as immune stimulators. The molecular variety of the solCR system is therefore further complicated by its functional diversity.

The existence of different isoforms for each solCR, as well as soluble checkpoint receptor-ligand complexes, create obvious challenges with detection and interpretation of quantification data. When measuring solCRs of the same name, different commercial kits seem to provide only partially overlapping data. It is currently unknown if the molecular species measured are individual or multiple isoforms of the same solCR, or molecular complexes between soluble receptor and soluble ligand, but the implications are significant, because assigning specific biological effects to the correct isoform and disentangling receptor-specific or ligand-specific quantifications would have important consequences for the interpretation and the understanding of the underlying biological processes. This calls for the establishment of standardised measuring criteria for these molecules, as well as a more open comparison of antibody clones and molecular epitope details between manufacturers.

Incidentally, findings from a previous publication on the immunological correlates of acute hepatitis C virus (HCV) infection (9) revealed the relevance of being able to distinguish and measure differential isoforms of immune soluble factors, how this can explain disease progression, and how this may offer novel suggestions for therapeutic targets. In that paper, two competing isoforms of the T-cell chemoattractant CXCL10 were measured, a full-length agonist and a truncated physiological antagonist, in a well-characterised longitudinal cohort of individuals with acute HCV infection, and it was discovered that chronic progression of infection was associated with increased systemic levels of the truncated antagonist isoform (presumably leading to loss of T-cell recruitment to the liver and failure to clear the virus), compared to low basal antagonist levels measured in patients who spontaneously resolved infection (9). Notably, a case study published after that paper (10) revealed that preventing truncation of CXLC10 could reduce HCV viral load (10). However, because this was an individual case report, it is difficult to determine if other confounding factors may have influenced the described outcome.

To conclude, a thorough investigation of the solCR system and its complexities will undoubtedly benefit both basic and translational research, by providing a better understanding of how truly informative these molecules can be as disease biomarkers as well as how effective they may be as pharmacological targets or immunotherapeutic agents.

## Author contributions

The author confirms being the sole contributor of this work and has approved it for publication.
